# All-optical transistor- and diode-action and logic gates based on anisotropic nonlinear responsive liquid crystal

**DOI:** 10.1038/srep30873

**Published:** 2016-08-05

**Authors:** Cheng-Yu Wang, Chun-Wei Chen, Hung-Chang Jau, Cheng-Chang Li, Chiao-Yu Cheng, Chun-Ta Wang, Shi-Ee Leng, Iam-Choon Khoo, Tsung-Hsien Lin

**Affiliations:** 1 National Sun Yat-sen University, Department of Photonics, Kaohsiung, 804, Taiwan; 2 Pennsylvania State University, Electrical Engineering Department, University Park, PA 16802, United States

## Abstract

In this paper, we show that anisotropic photosensitive nematic liquid crystals (PNLC) made by incorporating anisotropic absorbing dyes are promising candidates for constructing all-optical elements by virtue of the extraordinarily large optical nonlinearity of the nematic host. In particular, we have demonstrated several room-temperature ‘prototype’ PNLC-based all-optical devices such as optical diode, optical transistor and all primary logic gate operations (OR, AND, NOT) based on such optical transistor. Owing to the anisotropic absorption property and the optical activity of the twist alignment nematic cell, spatially non-reciprocal transmission response can be obtained within a sizeable optical isolation region of ~210 mW. Exploiting the same mechanisms, a tri-terminal configuration as an all-optical analogue of a bipolar junction transistor is fabricated. Its ability to be switched by an optical field enables us to realize an all-optical transistor and demonstrate cascadability, signal fan-out, logic restoration, and various logical gate operations such as OR, AND and NOT. Due to the possibility of synthesizing anisotropic dyes and wide ranging choice of liquid crystals nonlinear optical mechanisms, these all-optical operations can be optimized to have much lower thresholds and faster response speeds. The demonstrated capabilities of these devices have shown great potential in all-optical control system and photonic integrated circuits.

There have been intense efforts in researching and developing *all-optical* analogues of electronics in order to circumvent limitations associated with electronic devices as well as to fulfill the need for such optical elements in integrated photonic circuits. Unlike electronic devices, where the control of electronic processes can be effected by almost any materials with the application of voltages or fields, control of one light beam by another in all-optical processes is mediated by light induced changes in a nonlinear optical materials. Since the magnitude and response speed of the underlying nonlinear mechanisms vary very widely, interests and motivations for all-optical studies also vary greatly^1^,^2^,^3^,^4^,^5^,^6^,^7^,^8^,^9^,^10^,^11^,^12^,^13^,^14^,^15^,^16^,^17^,^18^,^19^. Some studies emphasize the speed performance, while others stress the optical threshold power/energy required or the application wavelength bandwidth. While one category such as all-optical diode and transistor actions may stress the fundamental interests and potentials, other studies on all-optical filters, switches, and limiters concentrate on how these all-optical processes may be implemented in practical devices. In general, what are regarded as newsworthy or high performance in one category tends not to be the case in the other category. For example, single photon transistors and molecular switches which are attractive from fundamental standpoints require impractical cryogenic (approaching absolute zero) operating temperature^9^,^10^,^11^,^12^. Others that do function at reasonable ambient temperature incur costly material processing or cumbersome fabrication procedure^12^,^13^ or, in the case of spatial soliton in nematic liquid crystals (NLC)^15^, involves tedious optical manipulation and critical balance of the input laser beam shape and intensity^15^.

In this paper, we show that anisotropic photosensitive nematic liquid crystals (PNLC) made by incorporating a small concentration of anisotropic absorbing dyes in nematic liquid crystals are promising candidates for constructing active all-optical elements by virtue of the extraordinarily large optical nonlinearity of the nematic host. In particular, we will demonstrate several ‘prototype’ devices such as optical diode, optical transistor capable of cascadability, fan-out and logic-level restoration as well as primary logic gate operations (OR, AND, NOT) based on such optical transistor. An important point to note is that these operations can all be realized at room temperatures.

In general, NLC’s possess both ultrafast individual molecular optical nonlinearities that response in the sub-picoseconds time scale, and slower macroscopic crystalline optical nonlinearities with milliseconds–nanoseconds speed^16^,^17^,^18^. In the context of optical induced refractive index change, the so-called optical Kerr effect, the magnitude of the refractive index coefficient n_2_ [defined by the index change Δn per unit laser intensity I] characterizing the ultrafast individual molecular electronic processes of NLC is on the order of 10^−14^ for non-resonant NLC to nearly 10^−11^ cm^2^/W for chiral NLC (more often called cholesteric liquid crystal), due to enhancement by photonic bandgap/band-edge dispersion^19^,^20^. Collective/crystalline optical nonlinearities are due to laser induced order parameter change [by trans-cis isomerization of azo-dye dopant, or the NLC itself can be an azo-compound], or simple laser heating through absorption, or purely dielectric interaction that causes crystalline [director axis] reorientation^16^,^17^. These can response anywhere from 10’s of nanoseconds to many milliseconds, depending on the temporal characteristics [cw or pulsed] and the intensity of the impinging lasers, with characteristic n_2_ that can range over several decades [~10^−4^ cm^2^/W to well over 1000 cm^2^/W]. Generally, the larger is the nonlinearity, the slower is the response time. This trade-off between speed and nonlinearity (which determine the power thresholds) in the case of nematic liquid crystals actually does allow one to determine a sizeable regime for performance that is competitive with other classes of nonlinear optical materials^16^,^21^. Another attractive property of nematic liquid crystals is that in general, because of the extraordinarily large nonlinearity, the interaction length needed to trigger the nonlinear optical processes are in the microns range^20^ and thus allow integration of NLC in compact photonic circuits/platforms, due also to their fluidic nature and compatibility with all technologically important materials^16^,^21^,^22^.

In this proof-of-concept study, we employ the laser induced order parameter change from dichroic dye-doped NLC in a twist-alignment (TN) configuration^23^,^24^, c.f. Fig. 1a. NLC in twist alignment is commonly employed in electro-optical display devices. If the incident polarization is set either perpendicular or parallel to the director axis on the entrance boundary surface, it will follow the rotation of the director axis provided that the conditions 2d·Δn >> λ is satisfied, where Δn is the birefringence, d the thickness and λ the wavelength (Mauguin theorem). Switching action (of the low-level light in electro-optical display devices) is performed by an applied AC electric field between the two transparent electrodes coated cell windows that enclose the NLC; the applied field causes a reorientation of the birefringent NLC and therefore changes the polarization state of the light after traversing the NLC, and consequently the transmission properties of the whole polarizer + NLC + polarizer assembly. All-optical switching is achieved by exploiting the intensity dependent birefringence through laser induced order parameter changes in the NLC. At low optical powers, the NLC is not perturbed and the twist-alignment will rotate the input polarization to exit as an orthogonally polarized light and hence it is blocked by the exit polarizer. When the optical power reaches a level high enough to reduce the order parameter [e.g. by trans-cis isomerization or absorption-heating of dye dopants], it would cause the birefringence to decrease and negate the rotatory action on the optical field polarization vector; some light would begin to get through. In the completely disordered state, the incident light retains its original polarization state and is fully transmitted as show in Fig. 1a. Similar configurations has been used for efficient all-optical Fourier image processing^24^.

## Results and Discussion

We begin by constructing an optical diode and evaluate its forward/backward transmission characteristics, and input/output isolation performance. We then designed an optical transistor with single wavelength pump-probe system, and demonstrate cascadability, fan-out and logic-level restoration. Finally, all optical logic gate (OR, AND, NOT) operations based on such optical transistor are demonstrated.

### Optical diode

**Figure 1:**
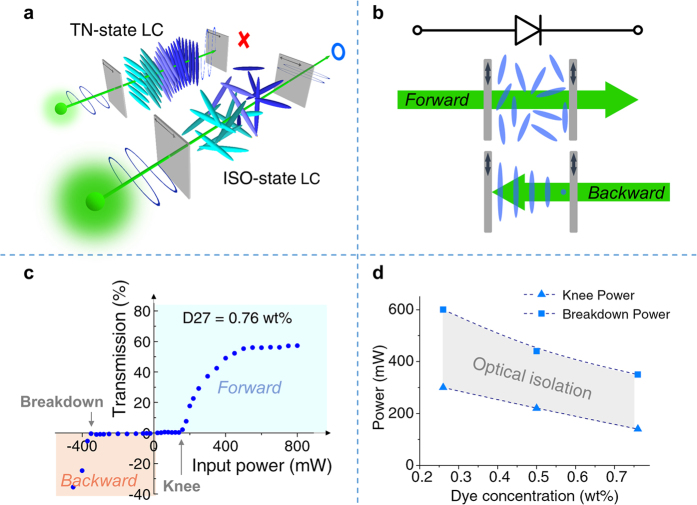
The dye-doped TNLC optical diode. The schematic diagrams of (**a**) TNLC-based optical switch (**b**) TNLC-based all-optical diode. Its measured (**c**) diode characteristic curve, and the (**d**) optical isolation region of the device under different concentrations. Operation temperature was controlled at 25 °C to minimize the thermal fluctuations from environment.

Diode is a basic component of an electronic transistor. The optical equivalence is a passive element possessing non-reciprocal transmission, i.e., it transmits light in one direction and blocks the reverse counterpart. As depicted in Fig. 1b, forward bias is defined as the direction in which an optical signal comes from the substrate with its alignment axis parallel to the transmission axis of the polarizer, whereas backward bias is the reverse direction. Under forward bias, at low input powers, the polarization state of the incident signal follows the twisted director axis of the LC rotating by 90° and subsequently terminated by the rear polarizer. Optical diode action begins when the input power is high enough (i.e. over the ‘knee’ power *P*_knee_) to cancel out the polarization-rotation effect by photo-induced order parameter change that reduces the local nematic birefringence. The transparency reaches its maximum value when the TNLC becomes isotropic. Under backward bias, the transmittance remains near zero when increasing the input pass *P*_knee_ because of poor absorption since the polarization is perpendicular to the absorption axis of the TNLC. However, at a very high power input (which may be termed the ‘breakdown’ power *P*_b_), the absorption could be appreciable to affect the order parameter change, and the backward-biased diode would then permit a fraction of light to pass through. The operation range for the TN optical diode action is therefore from *P*_knee_ to *P*_b_.

Figure 1c shows the transmittance-input power (*T*-*P*) curve of the TN diode with dye concentration of 0.76%, mimicking the current-voltage (I-V) relation of a typical electronic diode. Under forward bias, the TN diode starts transmitting light as the input power is greater than 140 mW (*P*_knee_), eventually reaching the a maximum transmittance of ~57.2% at an input of ~500 mW. The maximum transmission is primarily dictated by the dye absorption loss; hence, reducing the doping level could improve the maximum transmission. Doping the TNLC with dye concentrations of 0.76 wt%, 0.5 wt%, and 0.26 wt% result in maximum transmittance of 57.2%, 70%, 78.5%, respectively. However, lower dye concentration results in a higher threshold power. For practical uses, the output power can be amplified by cascading the diode with an all-optical transistor that performs a signal magnification function (see section “Cascadability and fan-out”). Switching the propagation direction of the optical signal, the diode is opaque until the input power exceeds the breakdown threshold *P*_b_ at 350 mW. Due to the dichroic properties of D27, the *P*_b_ is more than twice as large as the *P*_knee_. This interval between *P*_knee_ and *P*_b_ represents the optical isolation region of the diode, where only the forward signals to pass through. The optical isolation region of the TN diode with different doping concentrations are also measured, c.f. Fig. 1d. The results show that the optical isolation interval is fully controllable by choosing the dye concentration properly. Higher doping level of D27 in 5CB exhibits stronger optical absorption, bringing about greater photo-thermal conversion efficiency and therefore lower values for *P*_b_ and *P*_knee_.

### All-optical transistor

**Figure 2:**
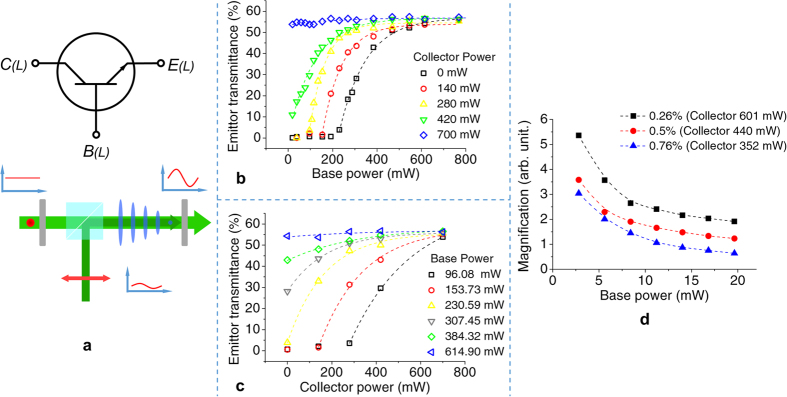
The dye-doped TNLC optical transistor. (**a**) The schematic diagram of a TNLC transistor. (**b**,**c**) Optical controllability (D27 concentration = 0.76 wt %) — relation between emitter transmission, collector power, and base power. (**d**) Fan-out — magnification factor with varying base powers, different colors stand for different dye concentrations.

By changing the self-modulation configuration to a tri-terminal (pump-probe) setup, the TN diode can function as an all-optical transistor [Fig. 2a]. The optical polarization ***E***_C_ of the collector (controlled) signal is set to be perpendicular to the absorption axis (long axis of the dye), while the base (controlling) signal has a polarization state ***E***_B_ parallel to the absorption axis. The modulated collector signal becomes the emitter signal having a polarization ***E***_E_//***E***_C_. By selecting appropriate driving powers *P*_B_ of the base to control the order parameter change of LC, the device yields controllable transmittance for the emitter signal.

Figure 2b compares the transmittance-to-base power (*T*-*P*_B_) curves under various collector powers *P*_C_ in the case of 0.76 wt% D27; here the transmittance is defined as the power ratio of the emitter signal to the collector signal. In the tri-terminal configuration, the base power controls the conversion efficiency between the collector and emitter powers. As the collector power is increased, the order parameter of the liquid crystal starts to change, and provides different initial working conditions for the transistor which is manifested in the decrease of the threshold base power value. With the same collector power, the device could be switched between transparent and opaque states by selecting the appropriate driving powers of the base, and the signal emitted from the transistor can be analogically/digitally modulated. The transmittance-to-collector power (*T*-*P*_C_) curves under various base powers *P*_B_ in the same device are depicted in Fig. 2c. Each curve represents the transmission properties of the transistor under the same driving base power. The increase of the base power lowers the threshold power of the collector, which fully demonstrates the optical control of the transistor. Similar to the diode case, the driving powers of the base can be adjusted by changing the doping level of D27, providing great flexibility for photonic circuit design.

### Cascadability and fan-out

Because the ON/OFF switching of the transistor is determined by the orientational order of liquid crystal in the transistor, the collector signal would directly pass through the device without distortions in the beam shape and wavelength (532 nm, in this study). Therefore, the emitter signal can act as the collector or base signal in the next stage. In a practical cascaded circuit, signal amplification is required to compensate the power loss generated in multiple stages. In our case, signal amplification is achieved by sending the weak signal into the base, and a strong light into the collector having its power set just below the *P*_b_. A clone of the base signal with enhanced power is thus generated and emits from the emitter. Figure 2d compares the performance of different transistors in signal magnification as characterized by the magnification factor M [defined as the power ratio between the emitter and base, P_E_/P_B_]. It shows that M ~3.58 can be achieved with the transistor at 0.5 wt% D27 for a collector power of 400 mW and base power of 2.805 mW at the ON state. This result implies that the device is able to support 3 subsequent stages. Since the value of M in Fig. 2d is inversely proportional to the baser power, higher magnification can be obtained at lower base power. For high magnification operation, it requires a high collector input (therefore lower efficiency) because the transistor cannot not be fully switched on by a low-power base signal. One possible means to improve the efficiency is to employ high dichroic ratio dye^25^.

### Logic-level restoration

**Figure 3:**
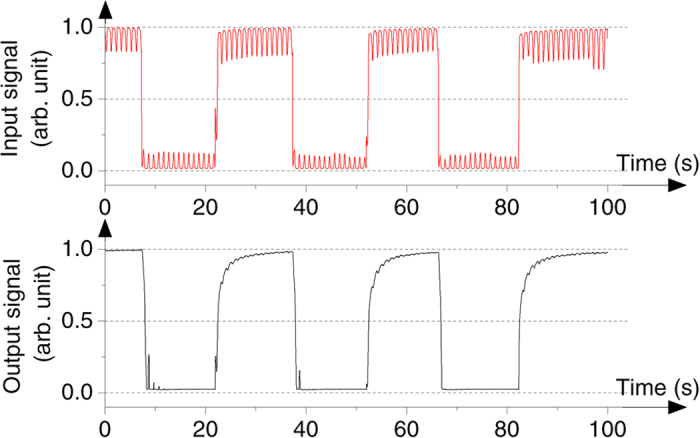
Logic level restoration. Red line is the pre-modulated base signal, and the black line is the output signal after logic restoration.

To enhance the performance of an optical logic circuit, an optical transistor should restore the signal perturbation to a certain logic level, so that there are only “OFF” and “ON” states in the circuit. Here we demonstrate the logic-level restoration ability of the transistor with 0.76 wt% D27. The collector signal is set at a power ~170 mW. The incident base signal is modulated into a square wave form (DC signal) with sine-shape perturbation (AC signal), as depicted in the red line in Fig. 3. Since the amplitude of the AC signal is 100 mW, which is lower than the *P*_knee_ value of base signal, a clear OFF state can be obtained. Further, the 700 mW of the DC offset gives rise to an optical isotropic state of the device, so that the fluctuation of the base signal is no longer able to influence the output performance of the ON state. Therefore, the logic-level restoration can be realized as shown in the black line of Fig. 3. To deal with different degrees of signal fluctuations, one can properly select the doping concentration of the dye. For example, by lowering the doping concentration, the device can withstand larger noises, leading to a higher perturbation tolerance in the OFF state.

### Logic gate

Here we demonstrate the possibility of using the transistor to perform fundamental logic-operations. Three fundamental operation modes- OR, AND, NOT can be achieved by properly adjusting the intensities of base and collector signals. The base signal is now separated into two identical laser beams for logic operation (herein, we term these two signals as A and B).

**Figure 4:**
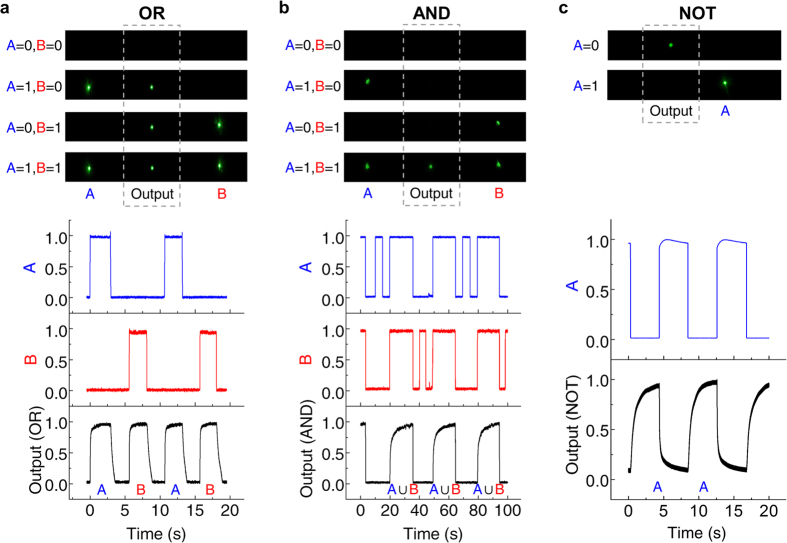
Logic gate operations (OR, AND, NOT). Upper parts are the spatial performance of the logic gates. The middle beam is the emitter signal, while that located at both sides represents the input of A and B signals. Lower figures plot the time domain performance when the transistor is operated under different logic modes. The black lines are the emitter signals and others are the base signals.

For an OR gate, the threshold power of the device should be less than either the power of A or B, while for an AND gate, the threshold power should be controlled between the power of A (or B) and A + B. As for the NOT gate (i.e., a converter), an opposite transmission behavior could be obtained by rotating the analyzer behind the transistor by 90 degrees. Figure 4 shows the time and spatial output response of the logic operations. These operations are realized by a TNLC transistor with 0.76 wt% of D27. In an OR gate operation, the device is operated at the collector power which is slightly lower than the *P*_b_ to keep the device off, and the power of both A and B are ~300 mW, thus the transistor can be turned on for each input signal. In an AND gate operation, both A and B signals are incident at a lower power of 75 mW, so that the transistor would only be turned on when the two pump signals input are present. A NOT gate is also successfully demonstrated. With the power of A set at ~300 mW, an OFF state of the transistor is produced once the base signal is on.

The performance of these all-optical diode, transistor and logic gate device operations are by no means optimized. There are still many factors that one can adjust to seek improvement, such as response time and power efficiency. For example, the threshold power of these device operations can be tuned with different dye concentrations. One could also use dyes with higher dichroic ratio^25^ and employ other nonlinear mechanisms^16^,^17^,^26^ to obtain faster response and lower threshold power.

## Summary

In this article, we have successfully demonstrated a room temperature all-optical diode and transistor devices by utilizing the nonlinear transmission property of dye-doped TNLC cell. Owing to the anisotropic absorption property of the AQ dye and the optical nonlinearity of the TNLC, spatially non-reciprocal transmission response can be obtained within a sizeable optical isolation region, e.g. *P*_b_ of 350 mW and and *P*_knee_ of 140 mW [isolation region *P*_knee_-*P*_b_ of ~210 mW]. Exploiting the same mechanisms, a TN-based transistor is demonstrated using a tri-terminal configuration as an all-optical analogue of a bipolar junction transistor. Its ability to be switched by optical fields enable us to realize the basic functions of an all-optical transistor, including cascadability, signal fan-out, logic restoration, and logical gate operations such as OR, AND and NOT. Due to the possibility of synthesizing anisotropic dyes and a wide choice of liquid crystals nonlinear optical mechanisms, these all-optical operations can be optimized to require much lower threshold and to response at higher speed. The demonstrated capabilities of these devices have shown great potential in all-optical control system and photonic integrated circuits.

## Method

The photosensitve NLC material is formulated by adding trace amount of a broadband-absorbing dichroic anthraquinone (AQ) dye, D27 (from BDH), into 4-Cyano-4′-pentylbiphenyl (5CB; from HCCH), a nematic LC having the clearing point at 35 °C and an intrinsic birefringence of 0.19 at 25 °C for *λ* = 532 nm; at 25 °C, the dichroic ratio of the mixture is ~10. In the experiments, samples with three different AQ-dye concentrations are used, namely 0.76, 0.50 and 0.26 wt%. The mixture is then introduced into a cell composed of a pair of polyimide-coated glasses with rubbing directions perpendicular to each other and separated with a gap of ~20 μm. Owing to the elastic continuum nature of LC, the NLC directors are twisted gently and continuously from one substrate to the other by 90°, thereby forming a twist-alignment NLC cell. Optical electric field (***E***, polarization) parallel to the transition dipole moment of the absorbing LC (***α***, usually parallel to the molecular axis) gives rise to more efficient absorption and order parameter change [e.g. by temperature rise or isomorphism] than the orthogonal counterpart polarization. This difference in photo-absorption by orthogonal polarizations equates to a marked difference between the threshold powers for the induced LC order parameter change associated with the two orthogonal input polarizations.

## Additional Information

**How to cite this article**: Wang, C.-Y. *et al*. All-optical transistor- and diode-action and logic gates based on anisotropic nonlinear responsive liquid crystal. *Sci. Rep.* **6**, 30873; doi: 10.1038/srep30873 (2016).
